# Host Cell Targets of Released Lipid and Secreted Protein Effectors of *Mycobacterium tuberculosis*

**DOI:** 10.3389/fcimb.2020.595029

**Published:** 2020-10-23

**Authors:** Jacques Augenstreich, Volker Briken

**Affiliations:** Department of Cell Biology and Molecular Genetics, University of Maryland, College Park, MD, United States

**Keywords:** *Mycobacterium tuberculosis*, effector, cell death, lipids, cytokines, phagosome maturation, NOX2, ESX-1

## Abstract

*Mycobacterium tuberculosis* (Mtb) is a very successful pathogen, strictly adapted to humans and the cause of tuberculosis. Its success is associated with its ability to inhibit host cell intrinsic immune responses by using an arsenal of virulence factors of different nature. It has evolved to synthesize a series of complex lipids which form an outer membrane and may also be released to enter host cell membranes. In addition, secreted protein effectors of Mtb are entering the host cell cytosol to interact with host cell proteins. We briefly discuss the current model, involving the ESX-1 type seven secretion system and the Mtb lipid phthiocerol dimycoserosate (PDIM), of how Mtb creates pores in the phagosomal membrane to allow Mtb proteins to access to the host cell cytosol. We provide an exhaustive list of Mtb secreted proteins that have effector functions. They modify (mostly inhibit but sometimes activate) host cell pathways such as: phagosome maturation, cell death, cytokine response, xenophagy, reactive oxygen species (ROS) response via NADPH oxidase 2 (NOX2), nitric oxide (NO) response via NO Synthase 2 (NOS2) and antigen presentation via MHC class I and class II molecules. We discuss the host cell targets for each lipid and protein effector and the importance of the Mtb effector for virulence of the bacterium.

## Introduction

Mtb is the bacterium responsible for the highest number of deaths annually caused by an infection disease. Its success as human pathogen is at least partially due to the ability of Mtb to evade the host immune response (Liu et al., 2017; Queval et al., 2017; Upadhyay et al., 2018; Bussi and Gutierrez, 2019; Sia and Rengarajan, 2019). On the other side there was intense selective pressure on humans to develop immune responses that could drive active tuberculosis infections into latent ones in order for the host to survive (Moreira-Teixeira et al., 2018; Olive and Sassetti, 2018; Simmons et al., 2018; Correa-Macedo et al., 2019). Ever since the first observation that intracellular Mtb inhibits the normal progression of phagosome maturations in the 1970s (Armstrong and Hart, 1971), the “How” and “What” of Mtb-mediated host cell manipulation have been under intense investigation. The translational research potential to exploit this knowledge, either for directly targeting Mtb effectors or for developing host targeted therapeutics, is important due to the urgent need of novel drugs to treat tuberculosis. Nevertheless, progress was limited for a long time due to the absence of genetic tools to modify Mtb. This changed in the early 1990s when pioneering work established the first tools to allow foreign gene expression in mycobacteria (Jacobs et al., 1987; Ranes et al., 1990) and to create a specific gene deletion mutant (Bardarov et al., 2002). Recently, ORBIT and CRISPRi technologies have been developed to simplify gene deletion/suppression approaches in Mtb (Rock et al., 2017; Murphy et al., 2018). The genetic toolbox was quickly expanded to include high-throughput gene disruption capacity via transposon (Tn)-mediated approaches which allowed for loss-of-function genetic screens (Camacho et al., 1999; Cox et al., 1999). The transposon approach was improved and combined with advances in gene sequencing technology to generate the TnSeq approach which allows for loss-of-function screens on a population basis (Long et al., 2015). Furthermore, shuttle cosmids were developed to be able to express large regions of the Mtb genome in non-tuberculoid mycobacteria in order to execute gain-of-function genetic screens (Bange et al., 1999; Velmurugan et al., 2007). Similarly, the genetic modification of host cells via siRNA and CRISPR/Cas9 allows for genome-wide genetic screens that can be combined with high-throughput readouts in order to identify host cell genes involved in specific host responses to Mtb infections (Kumar et al., 2010). The Collaborative Cross collection of mouse strains was used to identify host susceptibility and resistance genes following Mtb infections (Smith et al., 2019). These breakthroughs in experimental approaches have already led to seminal findings on Mtb lipid and protein effectors and their host cell targets. The goal of this review is to provide an overview of the current knowledge of secreted Mtb proteins and lipids, their mechanisms of action and their importance for virulence of Mtb.

## Mtb Effector Proteins and Their Targets in the Host Cell

What are some of the important characteristics of Mtb proteins that interact with the host cell? Many effectors need to be secreted by the bacterium and hence the secretion system for which they are a substrate is an important feature of the effector. The Mtb genome encodes for five type-seven secretion systems (T7SS), ESX-1 to ESX-5. They mediate the secretion of two family of proteins, EsxA-like proteins which are part of the Trp-X-Gly (WXG) family, and proteins harboring a Pro-Glu (PE) or Pro-Pro-Glu (PPE) N-terminal motif. The main substrates of ESX-1 are EsxA and EsxB. The ESX-3 system transports the EsxA/B paralogues EsxG/H. There is very little information on the substrates of the ESX-2 and ESX-4 systems. Finally, ESX-5 is responsible for secretion of a large number of proteins many of which are part of the PE/PPE superfamily (Gröschel et al., 2016; Bosserman and Champion, 2017). The heterologous expression of PE/PPE proteins in *M. smegmatis* (Msme) is a commonly used approach to characterize these proteins since they are absent in this mycobacterial species. Nevertheless, Msme also does not express an ESX-5 system, so the transport of these heterologously-expressed Mtb proteins is unknown. In order to focus the scope of this review we have decided to not include a discussion of Mtb PE/PPE effectors expressed in Msme, unless there is additional experimental evidence derived from Mtb deletion mutant or ectopic expression of the Mtb protein in a host cell. Furthermore, we also decided to limit the review to proteins that are secreted and released by Mtb which excludes many PE/PPE family proteins because they remain associated with the Mtb cell wall (e.g., PE-PGRS33). We point the reader to a current and exhaustive review on PE/PPE Mtb proteins (De Maio et al., 2020). In the absence of experimental data, we used SignalP 5.0 (Almagro Armenteros et al., 2019) to predict the presence of a signal peptide which indicates secretion via the SecA1/2 or Tat secretion systems.

Another important characteristic is the function of the effector. Protein tyrosine phosphatases A and -B (PtpA, PtpB) and the secreted acid phosphatase of Mtb (SapM) are the only three phosphatases known to be secreted by Mtb (Koul et al., 2000; Saleh and Belisle, 2000) (for more detailed review see Wong et al., 2013). The Mtb genome encodes 11 serine/threonine protein kinases of which 9 have a single transmembrane domain, an extracellular sensor domain and the intracellular kinase domain (Av-Gay and Everett, 2000; Prisic and Husson, 2014). PknG and PknK have no transmembrane domain but only PknG has been shown to be secreted (Prisic and Husson, 2014). In addition, Mtb encodes for the tyrosine kinase PtkA which phosphorylates and activates the phosphatase PtpA (Zhou et al., 2015; Jaiswal et al., 2019). In addition to kinases and phosphatases there is a wide range of other enzymatic activities reported for effectors (Table 1) but these are not always associated with their function in host cell manipulation but rather with their primary function in Mtb homeostasis (e.g., echA2 Truong and Penn, 2020). Finally, for many effectors no specific activity has been determined yet.

**Table 1:** Summary overview of Mtb protein effectors.

**Name**	**Gene ID**	**Secretion pathway**	**Function**	**Host target**	**Host cell process**	**Impact of gene deletion on Mtb virulence**
PtpB	Rv0153c	?	Phosphatase	?	?	Attenuated *ex vivo* and *in vivo* (guinea pig)
HBHA	Rv0475	?	?		Apoptosis (A)	No effect *in vivo*
SodA	Rv3846	SecA2	Superoxide dismutase	Phagosomal Superoxides	Apoptosis (I)	Attenuated *in vivo*
Rv3654c	Rv3654c	Predicted TAT	?	PSF	Apoptosis (I)	Attenuated *ex vivo*
Rv3033	Rv3033	Predicted SecA1/2 SP	?	?	Apoptosis (I)	Attenuated *ex vivo* but no data for *in vivo*
GroEL2/HSP65	Rv0440	?	Chaperone	Mortalin	Apoptosis (I)	?
Eis	Rv2416c	?	lysine Nε-acetyltransferase activity	JNK	Apoptosis (I), Xenophagy (I), Cytokine response (I)	Not attenuated *in vivo*
MPT53/DsbE	Rv2878c	Predicted SecA1/2	Disulfide oxidoreductase	Tak1	Cytokine response (A)	Hypervirulent *in vivo*
PPE13		ESX-5	?	NLRP3	Cytokine response (A)	?
EchA1	Rv0222	?	Probable enoyl-CoA hydratase	SHP1, TRAF6	Cytokine response (I)	Attenuated *in vivo*
EsxA	Rv3875	ESX-1	?	TLR-2, SR-B1, B2M	Cytokine response (I), Antigen presentation(I), Invasion(A), Pore formation (A)	Attenuated *ex vivo* and *in vivo*
Hip1	Rv2224c	Predicted SecA1/2	Esterase and Protease activity	GroEL2 (a secreted Mtb protein!)	Cytokine response (I), Apoptosis (I)	Attenuated *ex vivo* and *in vivo*
LpqN	Rv0583c	Predicted SecA1/2	?	CBL	Cytokine response (M)	Attenuated *ex vivo* and *in vivo*
CpnT/TNT	Rv3903c	?	hydrolyses NAD+	NAD+	Necrosis (A)	Not attenuated *in vivo*
PPE2	Rv0256c	ESX-5?	Transcriptional repressor	inos gene promotor, p67phox	NO and ROS production (I)	Attenuated *ex vivo*
SapM	Rv3310	SecA2	Phosphatase	Phosphatidyl-inositol-3-phosphate	Phagosome maturation (I)	Attenuated *ex vivo* and i*n vivo* (guinea pig)
PknG	Rv0410c	SecA2	Serine/Threonine kinase	Rab7L1/Rab29	Phagosome maturation (I)	Attenuated *ex vivo* and *in vivo*
CpsA	Rv3484	?	?: contains LCP and LytR domains	?: inhibits NOX2 activation	Phagosome maturation (I)	Attenuated *ex vivo* and *in vivo* (mouse and zebrafish model)
TlyA	Rv1694	?	rRNA methylase, hemolysin	?	Phagosome maturation (I)	Attenuated *ex vivo* and *in vivo*
LpdC	Rv0462	SecA2	Lipoamide dehydrogenase	Coronin-1	Phagosome maturation (I)	Attenuated *in vivo* (probably due to role on metabolism)
EsxH	Rv0288	ESX-3	?	HRS	Phagosome maturation (I)	Attenuated *ex vivo* and *in vivo*
PE_PGRS30	Rv1651c	ESX-5	?	?	Phagosome maturation (I)	Attenuated *ex vivo* and *in vivo*
NdkA	Rv2445c	SecA2	GTPase Activation Protein (GAP)	Rab5, Rab7, Rac1	Phagosome maturation (I), Apoptosis (I)	Attenuated *ex vivo* and *in vivo* (SCID mouse model only)
PtpA	Rv2234	?	Phosphatase	VPS33B, Subunit H of V-ATPase, ubiquitin, GSK3	Phagosome maturation (I), Cytokine Response (I), Apoptosis (I)	Attenuated *ex vivo* not *in vivo*
Rv3364c	Rv3364c	?	?	Cathepsin G	Pyroptosis (I)	?
PE_PGRS47	Rv2741	ESX-5 ?, Predicted SecA1/2	?	?	Xenophagy (I)	Attenuated *ex vivo* and *in vivo*

*All listed proteins have been shown to be secreted. If data is available, the secretion mechanisms is indicated. If the secretion pathway is not yet determined, we used SignalP-5.0 to check for signal peptide prediction. The parentheses behind the host cell process indicate Activation (A), Inhibition (I) or Modulation (M). If not stated explicitly the virulence impact refers to mouse studies*.

One of the most interesting feature of a secreted effector is its host cell target (Table 1). The identification of a target can be fairly straight forward if there is a strong binding affinity which allows for co-immunoprecipitation or column-based enrichment approaches. Interactions that are of low affinity are much harder to characterize which is probably one reason that for many Mtb effectors their host cell targets have not been identified yet (Table 1). Related to the targeted host cell protein is the impact of the Mtb effector on the host cell signaling pathways. The manipulation of proinflammatory cytokine responses, phagosome maturation, autophagy and host cell death are the major pathways targeted by Mtb that have been identified today (Table 1). Since these host cell pathways are interconnected (for example, proinflammatory cytokine signaling may affect host cell death) a specific Mtb effector may affect more than one host cell signaling pathway.

Finally, what is the contribution of a given Mtb protein effector to the virulence of the bacterium? The answer to that question is complicated by many factors. One of them being the redundancy of Mtb effector proteins for a given host cell signaling pathway. For example, many proteins and some lipids target the maturation of the Mtb phagosome (Tables 1, 2). So, studying their individual effect on phagosome maturation will show meaningful differences but when taken into context of an infected mouse lung these differences observed during *ex vivo* infections may not be important enough to affect the overall survival of Mtb in the mouse lung. An approach to overcoming this hurdle would be by generating double or even triple Mtb gene deletion mutants. Another complicating factor is how to be sure if the observed *in vivo* effect of a given Mtb mutant is actually due to the lack of manipulation of the specific host cell signaling pathway and not instead due to some secondary function of the Mtb protein during *in vivo* infections. In the most extreme cases of moonlighting, Mtb effector proteins such as echA2 (Truong and Penn, 2020) this is obvious because of the known function of echA2 in bacterial homeostasis, but it is actually a concern for any Mtb deletion mutant used during *in vivo* infections. One way to address this question is to use a genetic approach by infecting wild type mice but also a knock-out mouse strain that is deficient in the host cell signaling pathway that the Mtb effector is targeting. If the knock-out mouse strain rescues the virulence of the Mtb mutant strain when compared to wild-type Mtb, it is fair to assume that the *in vivo* attenuation (or hypovirulence for that matter) is due to the specific host cell signaling pathway that the Mtb effector protein targets.

**Table 2:** Summary overview of Mtb lipid effectors.

**Name**	**Known release mechanism**	**Host target**	**Host cell process**	**Impact of gene deletion on Mtb virulence**
PIM, LM, LAM, ManLAM	Extracellular vesicles, shedding	TLR4, TLR2-TLR1-6, MR, DC-SIGN, Dectin-2	Cytokine response (A/I), Phagocytosis (A), phagosome maturation (I), cell death (A)	Essential, variability in acylations and LAM capping sugar moiety linked to the degree of virulence
TMM / TDM	Shedding	Mincle	Cytokine response (I), phagosome maturation (I)	Essential, but inability to form “cords” which is dependent on TDM attenuates the strains.
DAT/PAT	Extracellular vesicles, shedding	?	Cytokine response (I), phagosome maturation (I), cell death (A)	Decreased persistence *in vivo* in PDIM-deficient strains, decreased growth in human macrophages.
PGL	Extracellular vesicles, shedding	TLR2	Cytokine response (I)	Attenuation *in vivo*
PDIM	Shedding	?	Cytokine responses (I) phagocytosis (A), phagosome maturation (I), phagosome escape (A), autophagy (A) apoptosis (A), necrosis (A)	Attenuation *ex vivo* and *in vivo*
SL-1	Shedding	TLR-2	Cytokine reponse (I), Phagosome maturation (I), autophagy (I), cell death (A)	No attenuation *in vivo*, lack of coughing and transmission in guinea pigs
1-TbAd	Shedding	?	Phagosome acidification (I)	Attenuation in human macrophages

*The parentheses behind the host cell process indicate Activation (A), Inhibition (I) or Modulation (M). If not stated explicitly the virulence impact refers to mouse studies*.

## How Do Mtb Proteins Gain Access to the Host Cell Cytosol?

**Figure 1:**
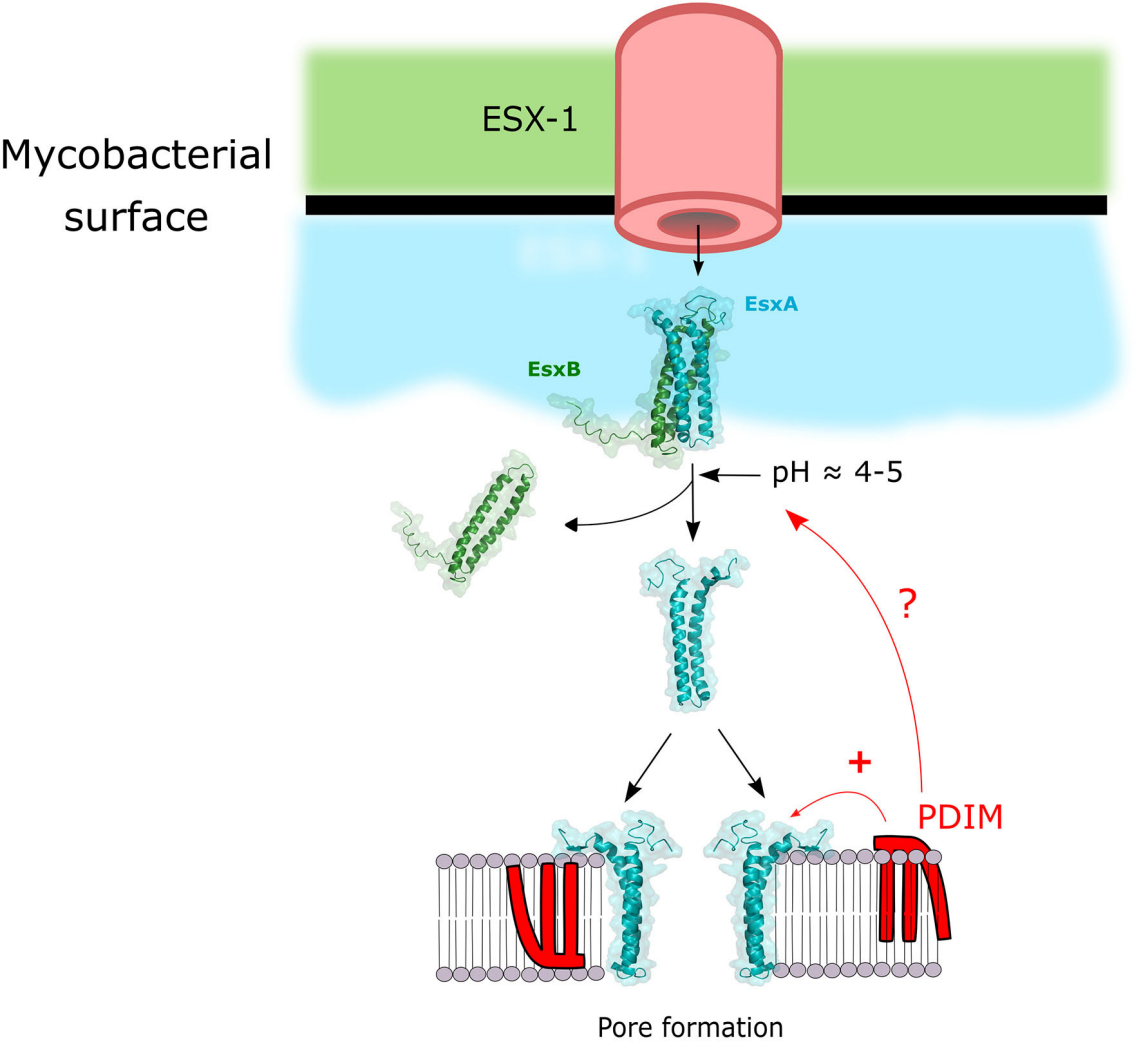
Model of the mechanism of membrane lysis by the cooperation of ESX-1 and PDIM. ESX-1 secretes EsxA and EsxB in a 1:1 heterodimer. This dimer is separated upon pH decrease and / or post-translational modifications, and free EsxA can induce pore formation. PDIM can potentiate EsxA membranolytic activity by either synergizing the pore forming activity of EsxA or by acting on the EsxA/B heterodimer complex separation.

In order for secreted Mtb proteins to reach host cell targets they have to overcome at least two barriers: the first one being the double membrane of Mtb (Hoffmann et al., 2008; Zuber et al., 2008) and the second one being the phagosomal membrane (Figure 1). The protein secretion systems of Mtb have been extensively reviewed elsewhere (Ligon et al., 2012; Majlessi et al., 2015). ESX-1 is arguably the most extensively studied member of T7SS in Mtb as its crucial importance in pathogenesis was described in numerous studies and reviewed extensively (Gröschel et al., 2016; Bosserman and Champion, 2017; Vaziri and Brosch, 2019). It is likely that the tremendous importance of ESX-1 for the virulence of Mtb is due to the fact that it allows Mtb effectors to gain access to the host cell cytosol by permeabilization of the phagosomal membrane (Figure 1). Gaining access to the cytosol via phagosomal membrane permeabilization in order to target host cell targets with bacterial effectors is a successful strategy shared with many other intracellular pathogens (Kumar and Valdivia, 2009). The crucial virulence factor secreted by ESX-1 for the permeabilization process is EsxA (also named ESAT-6), which is secreted as a 1:1 heterodimer with EsxB (also named CFP-10) (Renshaw et al., 2005). EsxA membranolytic activity was first observed by Hsu et al. (2003). Since then numerous studies aimed to dissect the mechanism of action leading to membrane lysis (reviewed elsewhere Peng and Sun, 2016). But recently a study cast a doubt on published EsxA *in vitro* studies suggesting that traces of detergent left from the purification process were responsible for the membranolytic activity observed (Conrad et al., 2017). Nevertheless, some studies were performed with detergent-free purification process and showed an EsxA pore-forming activity on membranes (de Jonge et al., 2007; Ma et al., 2015; Zhang et al., 2016; Ray et al., 2019; Aguilera et al., 2020; Augenstreich et al., 2020). Regardless of this confounding issue, there is good agreement that one crucial step for the lytic activity of EsxA is the separation of EsxA from EsxB after the secretion of the heterodimer through the ESX-1 system. That process can be mediated through an acidification that can lead to EsxA release and membrane binding (de Jonge et al., 2007). In contrast, other reports show that the membrane lysis process seem to happen at a mildly acidic pH and Mtb could rupture the phagosomal membrane even after a bafilomycin treatment which inhibits phagosome acidification (Simeone et al., 2015; Augenstreich et al., 2017). Hemolysis studies also suggested that a RD1-mediated lysis can happen at pH7 (Smith et al., 2008; Conrad et al., 2017; Augenstreich et al., 2020). Thus, the separation of the EsxA/EsxB complex seems to take place at neutral pH. A new study started to unravel this mechanism by showing EsxA undergo acetylation through secretion that increases EsxA/EsxB separation by decreasing the complex stability and contributes to *M. marinum* (Mm) phagosome escape (Aguilera et al., 2020). The lipidic virulence factor PDIM also showed to be essential for phagosomal escape during macrophages infection by Mtb (Augenstreich et al., 2017; Barczak et al., 2017; Quigley et al., 2017; Lerner et al., 2018). It is tempting to hypothesize that PDIM might also play a part in this process, that would explain the membrane lysis observed at neutral pH and that the lysis occurs at the contact point between the bacteria and the target membrane. But this will require additional studies to finally resolve the exact molecular mechanism of EsxA-mediated membrane lysis. Finally, even if EsxA is the main factor responsible for the lysis, other factors may be participating in the process, like the sphingomyelinase SpmT of Mtb (Speer et al., 2015) or uncharacterized ESX-1 associated factor(s) in Mm (Lienard et al., 2020).

It was long thought that the EsxA/ESX1-dependent phagosomal rupture was restricted to Mtb and Mm, since Msme despite encoding an ESX-1 system fails to escape the phagosome in macrophages. But recently it was described that *M. abscessus* was also able to rupture the phagosome, but through the use of ESX-4 (Laencina et al., 2018). Interestingly, *eccB4* deficient *M. abscessus* strains failed to inhibit phagosome acidification and to induce phagosomal rupture. It is linked to a secretion defect of the EsxA/EsxB-like complex EsxT/EsxU and one might speculate they act like their ESX-1 counterparts (Laencina et al., 2018). Thus, for *M. abscessus* that lacks an ESX-1 system, ESX-4 seems to perform the functions associated with ESX-1 in Mtb. In a potentially analogous mechanism to the EsxA/PDIM synergy described for Mtb, a new glycolipid was characterized in *M. abscessus* and its transport to the mycobacterial surface is required for phagosomal rupture (Dubois et al., 2018).

## Role of Mtb Proteins in Host Manipulation (Table 1)

### Phagosome Maturation

The uptake of Mtb by phagocytes generates a phagosome, the Mtb-containing vacuole (MCV). The normal maturation process of a phagosome is to fuse with early endosomes, then late endosomes and finally lysosomes to gradually acidify its lumen, acquire acidic protease and hydrolase in order to destroy the phagosomal bacterium (Upadhyay et al., 2018). One of the first immune evasion mechanisms assigned to Mtb was its capacity to prevent the fusion of lysosomes with the MCV (Armstrong and Hart, 1971). These excellent recent reviews provide additional information of Mtb-mediated inhibition of phagosome maturation (Upadhyay et al., 2018; Bussi and Gutierrez, 2019).

**EsxH**: The ESX-3 secretion system has a limited set of substrates comprised by EsxG-EsxH, PE5-PPE4, and the PE15-PPE20 heterodimers (Tufariello et al., 2016). EsxG and EsxH are EsxA and EsxB-like proteins respectively, which resolved in a 1:1 heterodimer structure is very similar to the EsxA/B complex but with a different function (Ilghari et al., 2011). A yeast two-hybrid (Y2H) screen identified the interaction of Mtb but not Msme EsxH with HRS (Hepatocyte Growth Factor-Regulated Tyrosine Kinase Substrate) (Mehra et al., 2013). HRS is important in the initial assembly of the ESCRT machinery which functions in transport of endosome to lysosomes for certain receptors and cargo (Szymanska et al., 2018). This interaction could be confirmed by co-immunoprecipitation experiments in HEK293 cells expressing EsxH and EsxG (Mehra et al., 2013). Importantly, the overexpression of EsxG/EsxH in Mtb increased the capacity of the bacteria to inhibit phagosome maturation (Mehra et al., 2013). The knock-down of HRS as well as downstream effector Tsg101 (one component of the ESCRT machinery) both resulted in a decreased MCV maturation showing that the host cell target of EsxH is functionally important (Mehra et al., 2013). It was subsequently shown that the inhibition of ESCRT by EsxH also reduced the capacity of macrophages and dendritic cells to present Mtb antigens and prime T-cells *ex vivo* and *in vivo* (Portal Celhay et al., 2016). Finally, ESCRT is recruited to the site of minor membrane damage and since Mtb, via ESX-1 and PDIM, permeabilizes the phagosomal membrane it was consistent with an ESX-1 dependent recruitment of ESCRT to the MCV (Mittal et al., 2018). Mtb is required to permeabilize the phagosomal membrane in order to manipulate the host cell. This damage can be recognized by the host cell ESCRT machinery which could result in increased phagosome maturation and antigen presentation. Hence, the bacterial adaptation to inhibit ESCRT-recruitment via secretion of its own effector EsxH. The importance of this system for Mtb virulence was demonstrated by the strong attenuation of an *esxH* deletion mutant in the mouse model with a 3–4 log reduction of CFU in the lungs (Portal Celhay et al., 2016; Tufariello et al., 2016).

**PE_PGRS30**: The ortholog of this Mtb gene in Mm (*mag* 24-1) is highly upregulated in bacteria present in the granuloma and its deletion results in loss of bacterial virulence *ex vivo* and *in vivo* (Ramakrishnan et al., 2000). Mag 24-1 is important for the capacity of the bacteria to inhibit phagosome maturation and exclusion of the vacuolar H^+^-ATPase from the MCV is a likely mechanism (Hagedorn and Soldati, 2007). The investigation of Mtb PE_PGRS30 led to very similar findings in regard to virulence and the importance of this gene for the capacity to inhibit phagosome maturation (Iantomasi et al., 2012).

**TlyA**: The TlyA protein has been shown to have rRNA methylase activity which functions in the methylation of 16S and 23R rRNA nucleotides (Johansen et al., 2006). Nevertheless, TlyA of Mtb also showed hemolysin activity when expressed in Msme (King et al., 1993; Wren et al., 1998). Consistently, purified TlyA can form oligomers on purified phagosomes and red blood cells leading to lysis (Rahman et al., 2010). TlyA peptides were identified in the culture filtrate of Mtb via mass spec analysis (Kelkar et al., 2011). The secretion of TlyA to the cell membrane does not require Tat or SecA2 secretion systems and TlyA is also included in membrane vesicles secreted by Msme (Kumar et al., 2015). In two gain-of-function approaches by expressing Mtb TlyA in Msme or coating latex beads with purified TlyA it could be demonstrated that TlyA mediates the inhibition of phagosome maturation by reducing Rab5, EEA1, and Rab7 recruitment to the phagosome but increasing Rab14 (Mittal et al., 2014). What the actual target of TlyA in the host cell is or how the phagosomal lytic activity connects to phagosome maturation inhibition remains to be determined. The Mtb TlyA mutant is attenuated in *ex vivo* infected macrophages and *in vivo* in BALB/c mice after aerosol infection but this study did not include a complemented mutant strain (Rahman et al., 2015).

**LdpC**: Is a lipoamide dehydrogenase which has an important function in the metabolization of branched-chain amino acids (Venugopal et al., 2011). Nevertheless, besides its cytosolic location and function, LdpC is also secreted via the SecA2 pathway (Zulauf et al., 2018). As such it was identified in the host cell cytosol to bind to host cell coronin-1 protein (Deghmane et al., 2007). This binding resulted in the retention of coronin-1 on the MCV membrane which inhibits phagosome maturation (Deghmane et al., 2007). The *ldpC* Mtb mutant is attenuated in the mouse model but this might be due to its function in Mtb metabolism which affects bacterial growth (Venugopal et al., 2011).

**PtpA**: PtpA is one of the three secreted phosphatases of Mtb (Koul et al., 2000; Saleh and Belisle, 2000). The tyrosine kinase PtkA of Mtb phosphorylates and activates cytosolic PtpA (Zhou et al., 2015; Jaiswal et al., 2019). Secreted PtpA affect three different host cell response pathways: (1) It binds to host cell ubiquitin which activates its phosphatase activity and leads to dephosphorylation of the host cell kinases JNK and p38 which reduces the pro-inflammatory cytokine response of the host cell (Wang et al., 2015a). (2) PtpA binds to and dephosphorylates host cell GSK3α which leads to less active caspase-3 and thus reduced host cell apoptosis (Poirier et al., 2014). (3) Mtb is able to inhibit recruitment of the host cell vacuolar-H^+^-ATPase (V-ATPase) to the MCV (Sturgill-Koszycki et al., 1994). PtpA binds subunit H of the V-ATPase (Wong et al., 2011) and it also binds to and dephosphorylates VPS33B (Bach et al., 2008), a protein enabling endosome to lysosome trafficking (Galmes et al., 2015). How these two capacities of PtpA are linked is not known but binding of PtpA to V-ATPase is a prerequisite for the dephosphorylation of VPS33B (Wong et al., 2011). The *PtpA* Mtb mutant is deficient in its capacity to inhibit the acidification of the MCV, inhibit phagosome maturation and growth in *ex vivo* infected macrophages (Bach et al., 2008; Wong et al., 2011). Nevertheless, the absence of PtpA does not alter growth of Mtb in the mouse model (Grundner et al., 2008). PtpA is phosphorylated on two tyrosines by the tyrosine kinase PtkA of Mtb which leads to activation of the phosphatase activity of PtpA (Zhou et al., 2015; Jaiswal et al., 2019). Consistently, the deletion of PtkA in Mtb leads to growth reduction in infected macrophages (Wong et al., 2018).

**SapM**: This phosphatase was identified via an elegant screen of Mtb culture filtrate fractions for acid phosphatase activity (Saleh and Belisle, 2000). It is secreted via the SecA2 pathway (Zulauf et al., 2018). SapM, unlike PtpA, has phosphatase activity on the lipid phosphatidylinositol-3-phosphate (PI3P) in addition to its tyrosine phosphatase activity (Vergne et al., 2005). The PI3P phosphatase activity of SapM is required to reduce the accumulation of PI3P on the MCV membrane which is key to inhibiting phagosome maturation (Vergne et al., 2005; Koliwer-Brandl et al., 2019). A point mutation abolishes phosphatase activity of SapM and the mutated protein is unable to mediate inhibition of phagosome maturation (Zulauf et al., 2018). A SapM deletion mutant is attenuated for *ex vivo* growth in macrophages (Saikolappan et al., 2012; Puri et al., 2013; Koliwer-Brandl et al., 2019) and *in vivo* growth in guinea pigs (Puri et al., 2013).

**PknG**: Mtb genome encodes 11 serine/threonine protein kinases of which 9 have a single transmembrane domain, an extracellular sensor domain and the intracellular kinase domain (Av-Gay and Everett, 2000; Prisic and Husson, 2014). PknG and PknK have no transmembrane domain but only PknG has been shown to be secreted (Prisic and Husson, 2014). The cytosolic location of PknG reflects the location of its many intracellular substrates (Baros et al., 2020) but PknG is also secreted via the SecA2 pathway (van der Woude et al., 2014; Zulauf et al., 2018). After infection of macrophages PknG can be found in the host cell cytosol where it mediates the inhibition of phagosome maturation and survival of bacteria (Walburger et al., 2004; van der Woude et al., 2014; Zulauf et al., 2018). The human RabGTPase protein Rab7L1 (Rab29 in mouse) is the host cell target of PknG (Pradhan et al., 2018). At the Golgi apparatus to PknG blocks the transition of inactive GDP-associated Rab7L1 to the active GTP-associated Rab7L1. This leads to the lack of recruitment of Rab7L1-GTP to the MCV which helps to inhibit phagosome maturation (Pradhan et al., 2018). The *pknG* Mtb mutant is attenuated in immunodeficient SCID mice and immunocompetent BALB/c mice after intra venous injections but not in the lungs of CD-1 mice after aerosol delivery (Cowley et al., 2004).

**NdkA**: The nucleoside diphosphate kinase A is secreted (Chopra et al., 2003; Målen et al., 2007) via the SecA2 pathway (Zulauf et al., 2018) and it seems to be an essential gene since it was not possible to generate deletion mutants neither in Mtb nor BCG (Sun et al., 2010, 2013). NdkA has GTPase activation protein activity which means that it accelerates the transition of small GTPase proteins from the GTP-bound (active) state to their GDP-bound (inactive) state (Chopra et al., 2004; Sun et al., 2010, 2013). Rab5 and Rab7 are small GTPase involved in vesicle trafficking and therefore also in the maturation process from early phagosome to late phagosome (Rab5) and late phagosome to phagolysosome (Rab7). Interestingly, NdkA isolated from Mtb but not NdkA from Msme can bind to Rab5 and Rab7 and dephosphorylate associated GTP. The *NdkA* knock-down strain of BCG is unable to inhibit the maturation process of the MCV (Sun et al., 2010). Another target of NdkA is the small GTPase Rac1 (Chopra et al., 2004; Sun et al., 2013). The inactivation of Rac1 results in a deficiency of the macrophages to assemble the functional NADPH oxidase (NOX2) complex on the MCV (Sun et al., 2013). The *NdkA* knock-down Mtb strain is inducing higher levels host cell ROS and host cell apoptosis. The strain is also attenuated for growth in *ex vivo* infected macrophages and in immunodeficient SCID mice (Sun et al., 2013).

### Cell Death

There are many different ways that a cell may die but for the purpose of this review we want to focus on apoptosis, necroptosis, and pyroptosis (Galluzzi et al., 2018). In the case of Mtb the current working model is that host cell apoptosis is detrimental for the virulence of Mtb, whereas host cell necrosis favors the pathogen (Behar et al., 2010; Srinivasan et al., 2014). It is thus not surprising that Mtb inhibits extrinsic and intrinsic apoptosis signaling pathways and has developed effectors to induces host cell necrosis (Moraco and Kornfeld, 2014; Srinivasan et al., 2014; Mohareer et al., 2018). The overall role of pyroptosis during the course of Mtb infections is not yet determined.

#### Pyroptosis

**Rv3364c**: The *Rv3361c-Rv3365c* operon is upregulated in Mtb after infection of macrophages and then the secreted Rv3364c can enter the host cell cytosol. Rv3364c binds to and inhibits expression and activity of Cathepsin G which leads to a reduced activation of the inflammatory caspase-1 and consequently less pyroptosis (Danelishvili et al., 2011).

#### Apoptosis

**Rv3654c**: This gene is expressed in an operon (*Rv3654c-Rv3660c*) which is upregulated after Mtb infection of macrophages. Rv3654c can reach the host cell cytosol where it binds to PSF (protein-associated splicing factor) (Danelishvili et al., 2010). The binding of Rv3654c to PSF leads to cleavage of the protein and reduced expression which results in reduced expression of caspase-8, a protease essential for signaling in the extrinsic apoptosis pathway (Danelishvili et al., 2010). Consequently, the *Rv3654c* Mtb mutant induced higher levels of TNF-mediated apoptosis and showed less survival during *ex vivo* infection of macrophages (Danelishvili et al., 2010).

**Rv3033**: A genome-wide screen for Mtb transposon mutants that have reduced survival in macrophages identified Rv3033 as important for survival in resting macrophages and macrophages treated with IFN-γ after infection (Rengarajan et al., 2005). Overexpression of Rv3033 in Msme conferred a reduction in host cell apoptosis (Zhang et al., 2018). The anti-apoptotic capacity of Rv3033 could be confirmed by expressing Rv3033 in a macrophage cell line and challenging with Mtb H37Ra infection (Zhang et al., 2018). Mtb Rv3033 targets the intrinsic, caspase-9 dependent, apoptosis pathway (Zhang et al., 2018). The deletion of Rv3033 reduces viability in *ex vivo* infected macrophages but no *in vivo* data is available (Rengarajan et al., 2005).

**SodA**: Superoxide Dismutase A is secreted via the SecA2-system and might be involved in the neutralization of superoxides generated in the MCV by the NOX2 phagocyte oxidase (Braunstein et al., 2003). The *SodA* gene is essential but an antisense inhibition of *SodA* expression in Mtb led to an important attenuation of the strain *in vivo*, marked by a high induction of apoptosis in the lungs of mice (Edwards et al., 2001). Later it was demonstrated that the absence of secreted SodA and the increase in host cell apoptosis *in vivo* leads to an increase in the presentation of Mtb-derived antigens (Hinchey et al., 2007).

**Eis**: The Enhanced Intracellular Survival protein was first identified in a gain-of-function screen of Mtb genes expressed in Msme that would increase survival of the bacteria after infection of macrophages (Wei et al., 2000). Eis is secreted by Mtb but it is unclear via which secretion pathway (Dahl et al., 2001) and it is able to reach the host cell cytosol (Samuel et al., 2007). The deletion of *eis* in Mtb causes an increase in host cell JNK kinase activation which leads to increased NOX2-mediated ROS generation, causing increased pro-inflammatory cytokine secretion, autophagy and host cell death (Samuel et al., 2007; Shin et al., 2010) The Mtb and Msme Eis proteins both have aminoglycoside N-acetyltransferase activity which confer resistance to antibiotics but on Mtb Eis also has lysine N^ε^-acetyltransferase activity (Kim et al., 2012). The latter activity of Mtb Eis targets acetylation of the host cell phosphatase DUSP16/MKP-7 which potentially increases its binding to JNK and inhibits its activation (Kim et al., 2012). The deletion of *eis* in Mtb does not reduce its virulence in mice (Samuel et al., 2007; Shin et al., 2010).

**Hip1 and GroEL2**: he Hip1 protein is likely cell membrane associated since it has a predicted lipoprotein signal peptide but one transmembrane domain predicted (Krogh et al., 2001; Almagro Armenteros et al., 2019) but was found in membrane and culture filtrate fractions (Målen et al., 2010; de Souza et al., 2011). Hip1 has esterase and protease activity (Naffin-Olivos et al., 2014) and GroEL2 is an Mtb chaperon that interacts among others with Mtb DnaK protein. GroEL2 is found in the cytosol and cell wall of Mtb and is one substrate for proteolytic cleavage by Hip1 which results in the release of a shorter protein into the supernatant (Rengarajan et al., 2008; Naffin-Olivos et al., 2014). New data shows that after Mtb infection GroEL2 gets released from the cell wall and can actually enter the host cell cytosol where ultimately it binds to the mitochondrial protein mortalin which has homology to Mtb DnaK protein (Joseph et al., 2017). Surprisingly, GroEL2 can access the host cell cytosol even in cells infected with the *M. bovis* BCG vaccine strain which lacks a functional ESX-1 system (Joseph et al., 2017). The interaction of GroEL2 with host cell mortalin mediates the inhibition of host cell apoptosis. There is no data available on the impact of GroEL2 deficiency on Mtb virulence, which is difficult to assess because GroEL2 is essential for *in vitro* growth of Mtb (Dejesus et al., 2017).

**HBHA**: eparin-binding hemagglutinin (HBHA) of Mtb is found in the cell wall and culture filtrate and is important of bacterial adhesion to epithelial cells but not macrophages (Menozzi et al., 1996). The deletion of *hbha* in Mtb results in a mutant strain that has similar virulence in the lungs of mice after intranasal infection but strongly reduced the ability for extrapulmonary dissemination as measured via CFU in the spleen (Pethe et al., 2001). Either heterologous expression of HBHA in Msme or the *hbha* Mtb deletion mutant demonstrated that this protein may lead to the increase in host cell apoptosis (Sohn et al., 2011). HBHA localizes to host cell mitochondria where it leads to increased activation of the pro-apoptosis protein BAX and increased levels of mitochondrial reactive superoxide generation (Sohn et al., 2011). The impact of increased host cell apoptosis during the context of *in vivo* infection in the lung is not significant (Pethe et al., 2001).

#### Necrosis

Mtb needs to escape its intracellular niche at some point in order to disseminate and infect other cells. Host cell necrosis favors pathogenesis of Mtb as has been shown by modulating host factors that tip the cell death modality toward necrosis instead of apoptosis (Behar et al., 2010). Host cell necrosis can actually stimulate the growth of Mtb (Dallenga et al., 2017; Lerner et al., 2017). In addition, the deletion of a transcriptional repressor (*Rv3167c*) resulted in an Mtb mutant strain that induced higher levels of host cell necrosis due to increased PDIM expression and was also hypervirulent in mice (Srinivasan et al., 2016). Despite its importance for virulence very little is known about Mtb effectors that induce host cell necrosis.

**CpnT/TNT**: The N-terminal domain of CpnT has pore forming capacity which is involved in uptake of small molecules through the mycomembrane (Danilchanka et al., 2014). The C-terminal domain (Tuberculosis necrotizing toxin, TNT) can be release after proteolytic cleavage and will target host cell coenzyme NAD^+^ for hydrolysis (Sun et al., 2015). The host cell depletion of NAD^+^ leads to necroptosis via the RIPK3/MLKL pathway but without activation of upstream signaling components such as RIPK1 (Pajuelo et al., 2018). The *cpnT* Mtb deletion mutant is not attenuated in mice (Danilchanka et al., 2014), suggesting that Mtb has redundant pathways for inducing host necrosis and most likely additional secreted effectors.

### Xenophagy

Xenophagy is a specialized form of canonical autophagy which results in the encapsulation pathogens by a double membrane autophagosome (Upadhyay and Philips, 2019). Xenophagy is a cell intrinsic defense mechanism against Mtb infection (Gutierrez et al., 2004). The ubiquitination of Mtb is dependent on the ESX-1 system and extracellular Mtb DNA (Watson et al., 2012). Ubiquitinated Mtb gets recognized by cytosolic autophagy receptors p62 and NDP52 which initiates autophagosome formation (Watson et al., 2012). The ubiquitin ligase Parkin 2 and Smurf1 are of critical importance for the ubiquitination of Mtb and host resistance to Mtb in the mouse (Manzanillo et al., 2013; Franco et al., 2016). Interestingly, Mtb expresses surface protein (Rv1468c) containing a eukaryotic-like ubiquitin-associated domain that binds ubiquitin and recruits p62 facilitating the xenophagic clearance of Mtb (Chai et al., 2019). Importantly, Mtb has yet to be defined mechanisms to inhibit host cell clearance via xenophagy as described for an ESX-1 dependent inhibition of autophagic flux (Romagnoli et al., 2012; Chandra et al., 2015; Cardenal-Muñoz et al., 2017). Please refer to following review for more background information (Khaminets et al., 2016; Upadhyay and Philips, 2019).

#### PE_PGRS47

This protein was identified in a gain-of-function screen using Msme for Mtb genes that mediated the inhibition of antigen presentation (Saini et al., 2016). Expression of PE_PGRS47 in Msme demonstrated that transport of the protein to the cell wall fraction (Saini et al., 2016) although EM studies in infected cells showed a location of PE_PGRS47 in the host cell cytosol (Saini et al., 2016). Msme does not express an ESX-5 secretion system and hence if it secretes PE_PGRS47 it has to be via a different secretion system which is somewhat surprising since ESX-5 is the major system for secretion of PE_PGRS proteins (Abdallah et al., 2009). The ability of PE_PGRS47 to limit antigen presentation is most likely indirect via its capacity to inhibit xenophagy and associated phagosome-lysosome fusion which results in the generation of Mtb peptides that can be presented at the cell surface (Saini et al., 2016). The PE_PGRS47 Mtb mutant is attenuated in immunodeficient and immunocompetent mice (Saini et al., 2016). The molecular mechanism and host target of PE_PGRS47 remain to be characterized.

### Cytokine Response

Host cell cytokines have important functions in host defense as demonstrated by the increased susceptibility of *tnf* ^−/−^ and *ifn*-γ^−/−^ mice to Mtb infections (Flynn et al., 1993, 1995). In contrast, IFN-β, a cytokine associated with anti-viral immunity, is exacerbating Mtb infections in mice and humans (Antonelli et al., 2010; Berry et al., 2010). Extracellular pattern recognition receptors such as TLRs and intracellular PRR such as NLRs are the sensors for pathogen associated molecular patterns (PAMPs) and after binding of a PAMP initiate a signaling cascade that leads to the production of cytokines. The importance of cytokines for host immunity to Mtb is reviewed in more detail in these excellent reviews (Mayer-Barber and Sher, 2015; Domingo-Gonzalez et al., 2016; Sia and Rengarajan, 2019).

#### Hip1and GroEL2

The interaction of Hip1 and GroEL2 have already been described in a previous section. The *hip1* Mtb deletion mutant induces increased pro-inflammatory cytokine production (TNF, IL-1β, IL-18, IL-6) in macrophages and dendritic cells via a TLR2/MyD88 signaling pathway (Madan-Lala et al., 2011, 2014). Importantly, just the overexpression of the cleaved and secreted GroEL2 fragment in the *hip1* Mtb mutant reverts the phenotype of the mutant back to wild-type Mtb, suggesting that the GroEL2 fragment is blocking the host cell TLR2/MyD88 signaling pathway (Naffin-Olivos et al., 2014). The *hip1* Mtb mutant is attenuated in *ex vivo* infected macrophages and *in vivo* infected mice (Rengarajan et al., 2008; Vandal et al., 2009) it is unclear to date though, if the attenuation *in vivo* is only due to the observed effect on GroEL2 or also due to the general susceptibility of the *hip1* mutant to low pH and ROS (Vandal et al., 2009).

#### PPE13

This member of the PE/PPE protein family contains a NxGxNxG motif which is characteristic for the major polymorphic tandem repeat (MPTR) subfamily of PPE proteins (Hermans et al., 1992). PPE13 does not contain a signal peptide and is secreted via the ESX-5 secretion system (Abdallah et al., 2009). Heterologous expression of PPE13 in Msme or ectopic expression in eukaryotic cells demonstrate that PPE13 activates the NLRP3 inflammasome leading to increased IL-1β secretion (Yang et al., 2020). Furthermore, PPE13 binds to NLRP3's NACHT and LRR domains via its MPTR domain (Yang et al., 2020). The PPE13-NLRP3 interaction facilitates homodimerization of NLRP3 and recruitment of the NLRP3 activator protein NEK7 (Yang et al., 2020). There is no data available on the impact of Mtb PPE13 on bacterial virulence.

#### LpqN

The lipoprotein has a signal peptide suggesting secretion via the SecA1/2 pathway and was found in the culture filtrate of Mtb (Målen et al., 2007). It was identified to bind CBL during a screening of 105 secreted Mtb proteins for host cell binding partners (Penn et al., 2018). CBL is a ubiquitin ligase that is increasingly phosphorylated after Mtb infections (Penn et al., 2018). The Mtb *lpqN* deletion mutant is growing less efficiently in *ex vivo* infected macrophages and *in vivo* (Penn et al., 2018). Importantly, the growth deficiency of the mutant in macrophages could be rescued by the deletion of host cell *Cbl* gene (Penn et al., 2018). The study shows data in support of CBL being a regulatorwhich suppresses anti-viral but supports anti-bacterial host cell intrinsic defense pathways; for example, *cbl*^−/−^ derived BMDMs are intrinsically more resistant to viral infection when compared to wild-type BMDMs. It is proposed that Mtb, by inhibiting CBL, induces an anti-viral host response which favors its own survival because anti-bacterial defense mechanisms are not induced (Penn et al., 2018).

#### EchA1

The enoyl-CoA hydratase A1 is involved in the lipid metabolism of Mtb but is also secreted via an unknown mechanism (no predicted signal peptide) and reaches the host cell cytosol (Wang et al., 2020). After ubiquitination of EchA1 by host cell ubiquitin ligase ANAPC2, EchA1 binds TRAF6 and SHP1 which prevents activation of TRAF6 and thus reduces the production of pro-inflammatory cytokines (Wang et al., 2020). The echA1 deletion mutant of Mtb is attenuated in the mouse model after aerosol infection (Wang et al., 2020).

#### PtpB

This is a broad-spectrum phosphatase that dephosphorylates phosphotyrosine, -serine and -threonine substrates in addition to various phosphoinositides (Beresford et al., 2007). Ectopic expression of PtpB in RAW264.7 murine macrophages conveyed inhibition of IFN-γ-mediated activation of the ERK1/2 and p38 signaling pathway toward increased IL-6 production and inhibition host cell apoptosis (Zhou et al., 2010). Nevertheless, these findings need to be confirmed via infection of cells with Mtb and a specific *PtpB* Mtb mutant. The deletion of *PtpB* resulted in a mutant that was less virulent in *ex vivo* macrophages infection models (Singh et al., 2003; Beresford et al., 2009; Koliwer-Brandl et al., 2019). Furthermore, the Mtb deletion mutant had an approximative 100 fold reduction in lung CFUs in the guinea pig model when compared to Mtb (Singh et al., 2003).

#### MPT53/DsbE

The protein is found in the culture filtrate (Målen et al., 2007) and has a predicted signal peptide (Almagro Armenteros et al., 2019) and is a disulfide bond-forming (Dsb)-like protein. In a screen of 208 secreted Mtb effectors expressed in HEK293T cells that changed NF-κB activation, DsbE was found to activate the NF-κB reporter gene (Wang et al., 2019). DsbE was found to bind to TGF-β-activated kinase 1 (TAK1) which is an important signaling molecule downstream of the TLR/TRAF6/TAB2 or TAB3 signaling pathway (Wang et al., 2019). TAK1 may activate NF-κB and the kinases JNKs and p38 which leads to the biosynthesis of pro-inflammatory cytokines (TNF, IL-6, IL-12). The binding of DsbE to TAK1 increased its phosphorylation which is required for activation (Wang et al., 2019). The enzymatic activity of DsbE is required for binding since a disulfide-oxidoreductase inactive mutant of DsbE fails to activate TAK1 (Wang et al., 2019). Consistent with this data the *dsbE* Mtb deletion mutant induced less TNF and IL-6 production in *ex vivo* infected macrophages and in the lungs of aerosol infected mice. The mutant was also hypervirulent in the mice with a 10 to 100-fold increase in lung CFUs after 21 d of infection (Wang et al., 2019). The fact that the secreted DsbE actually activates protective host responses suggest that its recognition by TAK1 is actually a host defense mechanism and the data that other Mtb and *E. coli* proteins with disulfide-oxidoreductase activity may also activate TAK1 supports this model (Wang et al., 2019). Thus, the sensing of bacterial disulfide-oxidoreductase activity in the host cell cytosol maybe a case of effector-triggered immunity (Lopes Fischer et al., 2020).

### NOX2/NOS2

The production of phagosomal ROS by the activated NOX2 and cellular NO by NOS2 are associated with cell intrinsic host defense (Bedard and Krause, 2007; Bogdan, 2015). Mtb is relative resistant to direct killing by ROS but the increase in phagosomal ROS observed after infection with *nuoG* and *secA2* Mtb mutants leads to an increase in host cell apoptosis which attenuates these mutants and leads to increased host cell antigen presentation (Hinchey et al., 2007; Velmurugan et al., 2007; Miller et al., 2010). The proteasome of Mtb is important for resistance of Mtb to killing via NO-derived reactive nitrogen intermediates (Darwin et al., 2003). Nevertheless, a more complex role for NOX2 and NOS2 during *in vivo* infections has emerged which associates them with a role in host immune tolerance (Olive and Sassetti, 2018).

#### CpsA

The protein was found in the culture filtrate (Målen et al., 2007) but has no predicted signal peptide sequence (Almagro Armenteros et al., 2019). A Y2H screen showed that CpsA binds to TAX1BP1 and NDP53 which are two proteins involved in xenophagy (Mehra et al., 2013), and SMCO1 (Penn et al., 2018). The use of knock-out host cells deficient in xenophagy, LC3-associated phagocytosis (LAP; see this reference for review Upadhyay and Philips, 2019) or both demonstrated that Mtb CpsA is involved in the inhibition of LAP (Koster et al., 2017). The precise host cell target of CpsA has not been determined but the exclusion of activated NOX2 from the nascent MCV is clearly an important aspect of the molecular mechanism of CpsA-mediated host cell manipulation (Koster et al., 2017). Overall, the end result is that a *CpsA* deletion mutant ends up in an MCV that fuses with lysosomes which results in decreased intracellular survival (Koster et al., 2017). The Mtb *CpsA* mutant is also attenuated in the mouse model (Koster et al., 2017; Malm et al., 2018) and deletion of the Mm homolog attenuated this pathogen in the zebrafish model (Wang et al., 2015b). The *CpsA* mutant induces increased ROS due to the activated NOX2 and it is thus likely that host cell apoptosis levels are also increased as this was shown before for several Mtb mutants that results in increased phagosomal ROS (Hinchey et al., 2007; Miller et al., 2010; Sun et al., 2013).

#### PPE2

This protein is secreted (Bhat et al., 2013) and since it is a member of the PE/PPE family it most likely is a substrate of the ESX-5 secretion system. PPE2 is targeted to the host cell nucleus via a nuclear location signal and binds to the promotor of the *Nos2* gene (Bhat et al., 2017). Consequently, infection of macrophages with the Mtb *Ppe2* deletion mutant and Msme overexpressing Mtb PPE2 result in increased or decreased NOS2 protein expression (Bhat et al., 2013, 2017). This activity resulted in increased survival of the Msme-PPE2 strain compared to Msme in *ex vivo* infected macrophages or *in vivo* infected mice (Bhat et al., 2017). PPE2 contains an SH3-like domain that allows for binding of the host cell p67phox NOX2 subunit. The binding inhibits p67phox recruitment to the phagosomal membrane and subsequent NOX2 assembly and activation (Srivastava et al., 2019). The overall result is that less ROS will be produced in the MCV which helps survival of bacteria during *ex vivo* macrophage infections (Srivastava et al., 2019).

### Multiple Host Cell Targets of Mtb EsxA

#### EsxA

**Figure 2:**
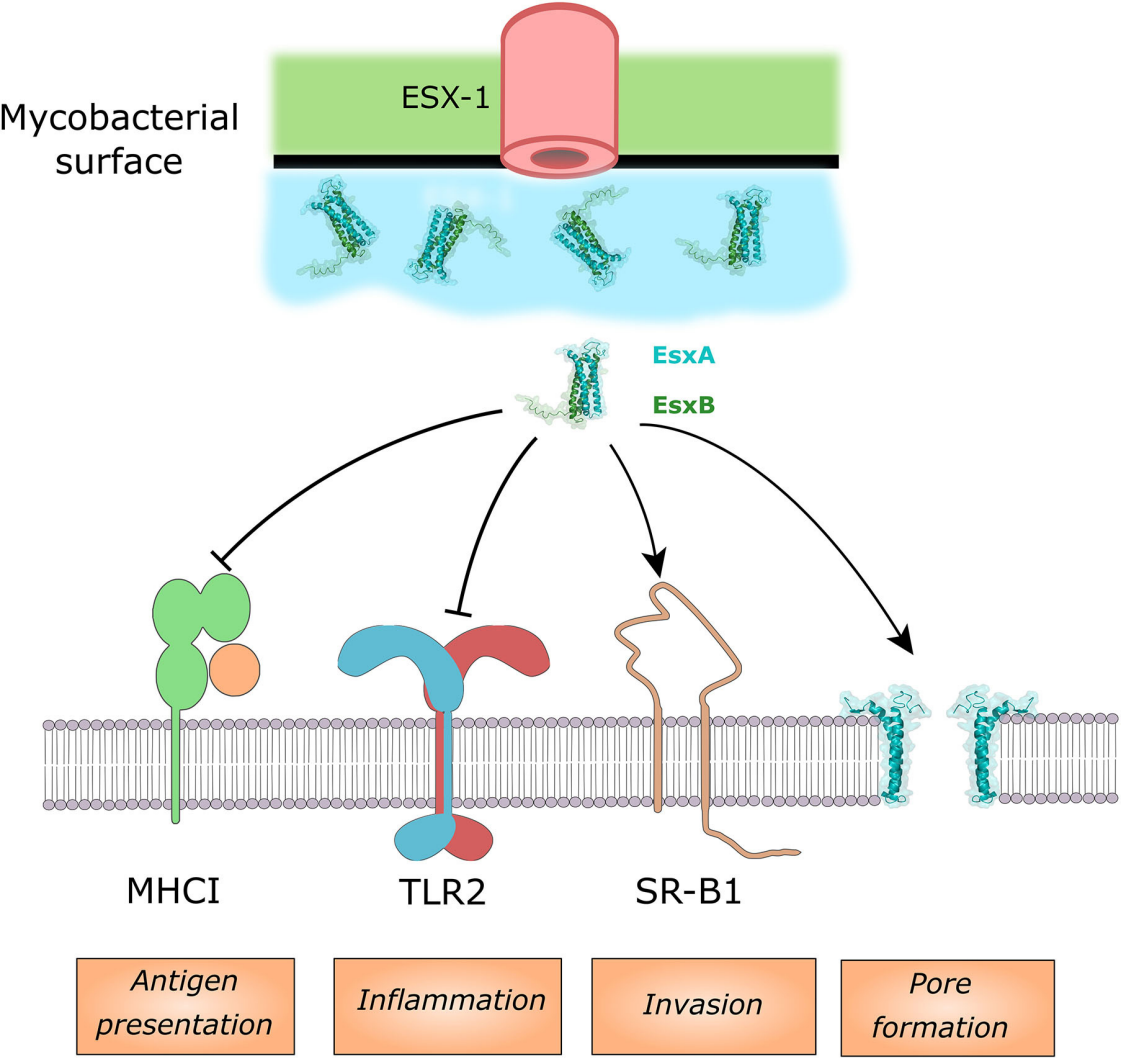
Host cell targets of EsxA and/or the EsxA/B complex. EsxA can antagonistically bind MHCI and TLR2, while it can bind to SR-B1 to enhance invading the lung epithelium. EsxA targets the phagosomal membrane for pore formation.

Due to the already discussed importance of EsxA on phagosomal membrane permeabilization it is challenging to experimentally dissociate phenotypes of the Mtb *esxA* mutant that are mediated by a direct effector activity of EsxA and those due to a lack of phagosomal membrane permeabilization and thus lack of access of other effectors to their host cell targets. Consequently, we focused this discussion on pathways in which a direct binding of EsxA to an effector protein could be shown (Figure 2), while acknowledging that other observed phenotypes of the Mtb *esxA* mutant might still be due to direct activity of EsxA. At the macrophages surface, EsxA inhibits TLR2 signaling by antagonistic binding to the receptor (Pathak et al., 2007). The inhibition of the downstream NF-κB pathway was also observed after incubation of cells with EsxA protein alone or in complex with EsxB (Ganguly et al., 2008). Both of these inhibitions lead to a decreased cytokine response by the infected host cell (Figure 2). EsxA can also interfere with antigen presentation to cytolytic T-cells by binding to the β2-macroglobuline which is associated with MHC class I. The binding of EsxA decrease the capacity of the MHC class I to present peptides due to decreased cell surface expression (Sreejit et al., 2014). A recent study found that EsxA can physically bind to the scavenger receptor B1 (SR-B1) and allow Mtb to cross the pulmonary epithelium through M cells (Khan et al., 2020). An Mtb-human protein-protein interactome screen identified several additional potential host cell binding proteins for EsxA but they require further validation (Penn et al., 2018). There have been many studies showing an impact of deletion of Mtb *esxA* on the host cell death response (apoptosis, necrosis, and pyroptosis) but in these studies it is difficult to discriminate between a direct or an indirect effect of EsxA.

## Mtb Lipids as Effector Molecules

**Figure 3:**
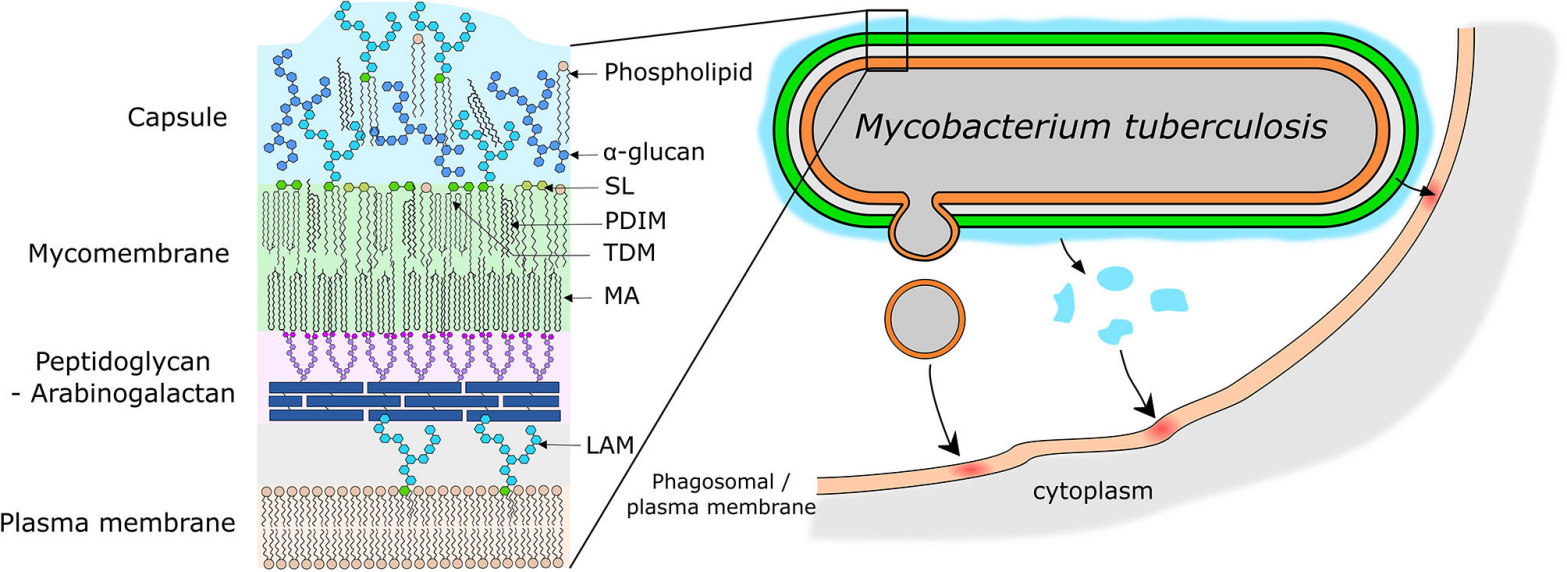
Models of mycobacterial envelope and lipid transfer. **(Left)** Simplified model of the organization of the mycobacterial envelope with the main virulence lipids highlighted. **(Right)** Representation of the different potential mechanisms of lipid release and transfer to the host cell membrane. Lipids can be release by emission of membrane vesicles, or by shedding of the capsular layer into the phagosome lumen or due to close physical contact directly into the phagosome membrane. SL, Sulfolipids; PDIM, Phthiocerol Dimycerosates; TDM, Trehalose Dimycolates; MA, Mycolic Acid; LAM, Lipoarabinomannan.

Mtb produces a wide variety of unique lipids which are important for host cell manipulation and virulence of Mtb (Neyrolles and Guilhot, 2011; Arbues et al., 2014; Gago et al., 2017; Queiroz and Riley, 2017). These lipids are localized in the mycobacterial envelope and have a very unique structure and role for pathogenesis (reviewed in Vincent et al., 2018; Dulberger et al., 2020, Figure 3). Briefly, the envelope consists of: (1) a plasma membrane which is mainly composed of phospholipids, (2) a superstructure made up of a layer of peptidoglycan covalently linked to arabinogalactan, and (3) a mycomembrane which as its inner leaflet has mycolic acids that are esterified with the underlying arabinogalactan (Figure 3). The outer leaflet of the mycomembrane is made up of a wide variety of lipids and almost all of them are involved in the host immune response manipulation by Mtb (Vincent et al., 2018; Daffe and Marrakchi, 2019; Dulberger et al., 2020) (Figure 3).

## How Do Mtb Lipids Get Into the Host Cell?

Mtb lipids are able to reach other organelles and the plasma membrane during infection providing evidence that some of the Mtb surface lipids could be released (Beatty et al., 2000; Rhoades et al., 2003). Mtb lipids are also found incorporated into exosomes and can thus reach uninfected bystander cells (Beatty et al., 2000; Athman et al., 2015). Thus, studying the way lipids are released by Mtb and their impact on the host is crucial to understand the pathogeny of Mtb. The section below summarizes the advances in the study of release of lipids by Mtb (Figure 3).

### Passive Release of Mtb Lipids

The first potential release mechanism is through direct contact of the bacteria with the host membranes, or by mild shedding of the capsular layer to which many lipids are loosely associated (Ortalo-Magné et al., 1996; Chiaradia et al., 2017). Indeed, in some phagosomes, mycobacteria are tightly surrounded by the phagosomal membrane and this feature was associated with the ability of *M. avium* to inhibit phagosome lysosome fusion (de Chastellier et al., 2009). Recent work showed an IFN-γ and Rab20 dependent increase of MCV volume which correlated with increased phagosome maturation (Schnettger et al., 2017). Moreover gold immunolabeling of Lipoarabinomannan (LAM) and phosphatidylinositol mannosides (PIM) suggested a transfer of these lipids from Mtb to the macrophages membranes through contact and/or release from the bacterial surface as the cryo sections did not show any vesicles emission (Beatty et al., 2000). Supporting these results, another structural study of Mtb found that the capsular layer can be removed through agitation of Mtb (Sani et al., 2010). A passive secretion followed by an insertion and diffusion supports their major impact on the immune response by also affecting bystander cells. PDIM, another major virulence lipid of Mtb, was found in macrophages membranes during infection (Augenstreich et al., 2019). Preliminary results on Mm coating with fluorescent modified PDIM indicated a transfer and a diffusion of these lipids upon contact with macrophages (Cambier et al., 2020). These results also support a passive transfer of PDIM upon contact of the bacteria with host cells. Such transfer by contact has also been observed for another bacterial species, *Borrelia burgdorferi*, where bacterial cholesterol-glycolipids were observed being transfer from the bacterial surface to the host cells plasma membrane (Crowley et al., 2013). Interestingly, the contact-mediated transfer accounted for 50% of lipid transfer, while the other part happened through membrane vesicles (MV) secretion by the bacteria (Crowley et al., 2013). Therefore, it is conceivable that for Mtb both contact dependent and independent transfers of lipids occur during infection (Figure 3).

#### Active Release of Mtb Lipids

The second mechanism of lipid release that has been recently described is through the release of MV (Prados Rosales et al., 2011) (for extensive review on this topic Brown et al., 2015; Layre, 2020) (Figure 3). These vesicles of ≈ 150 nm of diameter are apparently emitted by budding from the bacterial surface mostly observed in culture (Prados Rosales et al., 2011, 2014b; Athman et al., 2015), but production is also observed by bacteria localized in the host cell phagosome (Prados Rosales et al., 2011). Proteomics analysis indicated that some characterized antigenic factors were associated to the MVs also known for their inflammatory properties (Prados Rosales et al., 2011) and subsequent immunization of animals with Mtb MVs induced a protective immune response (Prados Rosales et al., 2014a). The lipid composition of the MVs is still only partially characterized, but phosphatidylinositol mannosides (PIM), lipoarabinomannan (LAM), poly-acyltrehaloses (PAT), and phenol glycolipids (PGL) (Prados Rosales et al., 2011). In addition, MVs include hundreds of Mtb proteins as determined by the host antibody responses to injected MVs (Prados Rosales et al., 2014a) and proteomics analyses (Prados Rosales et al., 2011). The PIM and LAM in MVs were associated to the lipid induced dampening of the acquired immune response by inhibiting T-cell activation (Athman et al., 2017). In contrast, some of these lipids were originally described as pro-inflammatory by activating TLR2 signaling in the case of lipomannan (Quesniaux et al., 2004). PGL are also present in MVs and they are also known as antagonist inhibition of TLR2 signaling (Arbues et al., 2016). The abundance of virulence lipids mainly present at the surface of the bacteria might indicate the other lipids like PDIM and trehalose dimycolate (TDM) that can diffuse out from the bacteria, may be present in the MVs but further investigations will be needed to attest of that. Also, the suggested lipid transfer from MV emission by Mtb to host cell membranes (Athman et al., 2015), still need to be formerly determined. The Mtb-produced MVs could potentially mediate immune modulatory effects beyond the site of infection and this might be of particular importance during the extracellular replication of Mtb within necrotic regions of human granulomas.

### Role of Mtb Lipids in Host Manipulation (Table 2)

The main effect described so far of the lipid panoply produced by Mtb is as inflammatory regulators (Table 2). An interesting “lipidic immunostat” model was proposed in which lipids are separated into pro- and anti-inflammatory (Queiroz and Riley, 2017). Indeed, the majority of the lipids produced are glycolipids whose sugar moieties are recognized by a wide range of pattern recognition receptors (PRR) (Ishikawa et al., 2017; Queiroz and Riley, 2017).

#### PIM, LM, LAM

This group of lipids is composed of lipids synthesized from phosphatidylinositol to generate the phosphatidylinositol mannosides (PIM) intermediates followed by the lipomannan (LM) and finally lipoarabinomannan (LAM) (Briken et al., 2004; Guerin et al., 2010; Sancho-Vaello et al., 2017). PIM (Gilleron et al., 2003) and to a lesser extent LAM/ManLAM (Nigou et al., 2008) are TLR2 ligands with PIM species acting as agonists, and LAM species acting as anti-inflammatory molecules (Quesniaux et al., 2004). LM is also a TLR2 agonist leading to cell signaling that induces IL-12 production and apoptosis (Dao et al., 2004). These lipids can therefore regulate TLR2/MyD88/NF-κB dependent production and secretion of numerous inflammatory cytokines such as TNF, IL12p40 or IL-8. A mannose-capped LAM (ManLAM) dampens the immune response through binding and inhibition of DC-SIGN signaling (Maeda et al., 2003). Alternatively, ManLAM can bind to Dectin-2 receptor, inducing an inflammatory response that appeared to be detrimental for mycobacteria in mice (Yonekawa et al., 2014). The mannose moieties of LAM can bind to the mannose receptor and stimulate the phagocytosis of Mtb (Maeda et al., 2003; Torrelles et al., 2006). Once in the bacteria are internalized, PIM (Vergne et al., 2004) and LAM (Fratti et al., 2001, 2003) contribute to the capacity of Mtb to inhibit phagosome maturation. PIM actually stimulates the fusion of the MCV with early endosomes which helps to avoid fusion with late endosomes (Vergne et al., 2004). The recruitment of the early endosome autoantigen (EEA1) protein to early phagosomes is an essential step in phagosome maturation that Mtb LAM is able to inhibit (Fratti et al., 2001). These excellent reviews provide a more in-depth overview on the activity of PIM/LM/LAM Mtb glycolipids (Vergne et al., 2014; Garcia-Vilanova et al., 2019).

#### TDM, DAT/PAT, SL-1

This group of Mtb lipids is composed of the trehalose-containing lipids (Garcia-Vilanova et al., 2019). Probably the best known lipid within this group is the essential lipid trehalose dimycolate (TDM), also called “cord factor” because it is required for the cording phenotype of Mtb which was originally described by Robert Koch in 1882 (Glickman et al., 2000). TDM binds to the host cell receptor Mincle and leads to macrophage and dendritic cell activation (Ishikawa et al., 2009; Ostrop et al., 2015) and also to the TDM-induced granuloma formation in the lungs of mice injected with TDM (Ishikawa et al., 2009). Inside macrophages, TDM contributes to enhance the survival of Mtb, as they are involved in phagosome maturation inhibition (Indrigo et al., 2003) and intracellular cording was recently associated to an inhibition of cytosolic detection of Mtb in endothelial cells, thus favoring persistence in lymphoid tissues (Lerner et al., 2020). Accordingly, Mtb mutants which have a deficiency of cycloprane modification in the mycolic acid chains of TDM show a defect in cording and a decrease in the granulomatous response *in vivo*, as well as the persistence in the host (Glickman et al., 2000; Rao et al., 2005).

A second group of compounds are the di- / poly-acyltrehaloses (DAT/PAT) (Garcia-Vilanova et al., 2019). They have no effect on virulence of Mtb in the mouse and guinea pig model (Rousseau et al., 2003a; Chesne-Seck et al., 2008; Passemar et al., 2014) but in the absence of PDIM a role in virulence could be detected for DAT/PAT in the mouse model (Passemar et al., 2014). At the cellular level DAT/PAT stimulate binding and entry of Mtb into macrophages and epithelial cells (Rousseau et al., 2003a). Mtb DAT/PAT are also important for the Mtb-mediated phagosome maturation inhibition (Brodin et al., 2010; Passemar et al., 2014).

The last member of this group is the Sulfoglycolipid-1 (SL-1) is only synthesized by Mtb and *M. canetti* and characteristically contains sulfated trehalose. Several studies demonstrated a role of SL-1 in the phagosome-lysosome fusion inhibition by Mtb (Goren et al., 1976; Brodin et al., 2010; Passemar et al., 2014). A Tn-mutagenesis genetic screen in Mtb identified Mtb mutants in genes involved in SL-1 biosynthesis to show increased activation of NF-κB after THP-1 cell infection when compared to Mtb (Blanc et al., 2017). Purified and synthetic SL-1 has antagonistic binding activity to TLR-2 which mediates decreased NF-κB activation, reduced pro-inflammatory cytokine production and costimulatory molecule expression (Blanc et al., 2017). In the same fashion, a report found that SL can inhibit autophagy through MyD88 signaling (Bah et al., 2020). SL-1 have no effect on virulence of Mtb in the mouse and guinea pig model (Rousseau et al., 2003b; Chesne-Seck et al., 2008). Recently, a study found a crucial role of SL-1 in the transmission process of Mtb by stimulating cough in guinea pigs (Ruhl et al., 2020). Indeed, SL-1 activates nociceptive neurons which triggers the coughing reflex and consistently, guinea pigs infected with a SL-1-deficient Mtb strain do not cough and do not transmit bacteria to uninfected guinea pigs (Ruhl et al., 2020).

#### PDIM, PGL

Another group is composed of the lipid DIM/PDIM and its glycosylated form the phenol glycolipids (PGLs). PDIM are only produced by pathogenic mycobacteria in the MTB complex (Goren et al., 1974; Vincent et al., 2018). PDIM-deficient strains are attenuated in the Guinea pig model (Goren et al., 1974), and these findings could be confirmed, two decades later, after a screening of a transposon mutant library of H37Rv for loss of *in vivo* virulence in the mouse model (Camacho et al., 1999; Cox et al., 1999). The attenuation of Mtb PDIM-deficient strains is more remarkable during the first weeks after infection suggesting a function in defense against the innate immune response (Rousseau et al., 2004; Murry et al., 2009; Kirksey et al., 2011; Day et al., 2014). At the cellular level, PDIM are anti-inflammatory lipids since a PDIM-deficient Mtb strain causes increased proinflammatory cytokines responses such as TNF and IL-6 in macrophages and dendritic cells (Rousseau et al., 2004). Consistent with this anti-inflammatory effect a PDIM-deficient Mm strain shows an increase in MyD88-dependent recruitment of macrophages to the granuloma in the zebrafish model (Cambier et al., 2014). Their presence also stimulates Mtb phagocytosis mediated by CR3 (Astarie-Dequeker et al., 2009), contributes to the phagosome maturation inhibition (Astarie-Dequeker et al., 2009; Passemar et al., 2014), modulates autophagic response (Bah et al., 2020), is required for phagosomal escape and cell death induction (Augenstreich et al., 2017; Barczak et al., 2017; Quigley et al., 2017). Studies of infection of human endothelial cells also showed that PDIMs are required for phagosomal escape (Lerner et al., 2018) and intracellular cording (Lerner et al., 2020). Presently, no host cell receptor for PDIM was identified, as the effect PDIM on CR3-mediated phagocytosis did not reveal any binding (Arbues et al., 2016).

PGLs are a glycosylated form of PDIM, with sugar moieties varying depending on the mycobacterial strain that produces the PGL (Arbues et al., 2014). The Mtb Beijing strains that are highly prevalent in Asia (Huet et al., 2009), *M.leprae* (Hunter and Brennan, 1981), Mm and *M. ulcerans* produce PGL lipid species (Daffé and Lanéelle, 1988). Mtb- or Mm-derived PGL have mostly an anti-inflammatory role by inhibiting inflammatory cytokines secretion (Reed et al., 2004; Robinson et al., 2008) and also contribute to phagosome maturation inhibition (Robinson et al., 2008). More recently it was found that Mtb PGL or its trisaccharide domain can bind to TLR2 and inhibit the NF-κB pathway (Arbues et al., 2016). Interestingly, in the zebrafish model the PGL of Mm are associated with a CCR2-mediated recruitment of permissive macrophages in order to increase virulence of Mm (Cambier et al., 2014).

#### 1-TbAd

The 1-tuberculosinyladenosine (1-TbAd) was discovered using an HPLC–mass spectrometry (MS)-based lipidomics approach and by comparing lipid profile of H37Rv and the *M. bovis*-derived vaccine strain BCG (Layre et al., 2014). This is a di-terpene linked adenosine lipid which was detected both associated on the bacteria and in the culture supernatant, suggesting a release of the lipid by shedding (Layre et al., 2014; Buter et al., 2019). This lipid was found in the vast majority of clinical isolates tested and appeared highly abundant (Buter et al., 2019). More interestingly, this lipid can act as an antacid when Mtb resides in a phagosome, so it can counter the decrease in pH due to phagosomal maturation (Buter et al., 2019). 1-TbAd can also diffuse out of the phagosome and induce swelling in lysosomes, thus inhibiting their fusion to the mycobacterial phagosome (Buter et al., 2019).

### Mtb Lipids as Modifiers of Host Membrane Biophysical Properties

In general, the role of lipids was mainly described as pathogen associated molecular pattern (PAMP). Nevertheless, their ability to transfer from the bacteria mycomembrane to the host cell macrophages membranes during the infection led to study their potential impact on host cell membrane structure and organization. For example, Mtb-derived ManLAM can disrupt microdomain (often called Raft) in artificial membranes and vesicles fusion (Hayakawa et al., 2007). They were also observed in these domains in ManLAM-treated cells and associated with a defect in phagosomes-lysosomes fusion (Welin et al., 2008). This property was also observed during Mtb infection of macrophages (Fratti et al., 2003). More recently, ManLAM was also found to bind to lactosylceramide in rafts at the plasma membrane and the phagosomal membrane, to induce phagocytosis and to inhibit phagosome-lysosome fusion, respectively (Nakayama et al., 2016).

An alteration of the membrane biophysical properties were also found with TDM inserted into artificial and isolated mitochondria (Sut et al., 1990; Harland et al., 2008) and they were able to decrease membrane fusion in liposomes model (Spargo et al., 1991). SL-1 increases membrane polarity on THP-1 treated with the purified lipid and that was associated with its autophagy inhibitory properties (Mishra et al., 2019; Dadhich et al., 2020).

PDIM lipids may not have a host cell receptor that mediates their effects because of their purely lipidic nature and it is thus conceivable their broad effects on macrophage responses are due to alterations in the membrane organization. Indeed, PDIM were found to decrease membrane polarity in macrophages infected with BCG (Astarie-Dequeker et al., 2009). Moreover, it was recently characterized that PDIM can adopt a conical shape in membranes that is responsible for an increase curvature of artificial membranes (Augenstreich et al., 2019). PDIM treatment of macrophages prior of infection can also rescue the phagocytosis level of PDIM deficient strain of Mtb. Interestingly, treating macrophages with the conical lipid Palmitoyl-Oleoyl Phosphatidylethanolamine can restore the phagocytosis of a PDIM-deficient strain of Mtb at the same level as a PDIM treatment (Augenstreich et al., 2019). This strongly suggested a tight link between PDIM conical shape and its effect on macrophages responses. All these observations on PDIM reveal that the biophysical impact of the insertion of Mtb virulence lipids into the host cells membranes is potentially underestimated for the other virulence lipids such as TDM, PGL, DAT/PAT or SL-1, and could explain part of the crucial importance the lipids play in Mtb virulence.

## Discussion

The knowledge of how Mtb effectors interact with the host cell has increased tremendously over the last decade and the successful application of system biology approaches to identify effector-host cell interactions has already revealed many new potential interactions that will certainly generate compelling new findings in the years to come (Mehra et al., 2013; Penn et al., 2018; Wang et al., 2020). We focused this review on proteins and lipids of Mtb that are secreted and released by the bacterium and affect host cell defense pathways. We certainly did not intend to diminish the importance of the other strategies of Mtb to manipulate the host cell: (1) Interactions of cell wall-anchored Mtb proteins and host cell membrane receptors (e.g., PE_PGRS33), (2) Mtb secretes nucleotides [c-di-AMP (Dey et al., 2015, 2017), Mtb DNA (Watson et al., 2012, 2015; Collins et al., 2015; Wassermann et al., 2015) and RNA (Cheng and Schorey, 2018)] that clearly interact with the host cell to; for example, induce IFN-β production in the case of secreted Mtb DNA (Collins et al., 2015; Wassermann et al., 2015; Watson et al., 2015) and (3) Mtb secretes membrane vesicles that contain cargo and include membrane bound lipids and proteins that will interact with the host cell (Prados Rosales et al., 2011; Brown et al., 2015; Lee et al., 2015).

## Author Contributions

JA and VB wrote and edited the manuscript and tables. JA created the figures. All authors contributed to the article and approved the submitted version.

## Conflict of Interest

The authors declare that the research was conducted in the absence of any commercial or financial relationships that could be construed as a potential conflict of interest.
